# Effects of Selective and Nonselective Beta Blockers on Bone Mineral Density in Mexican Patients with Breast Cancer

**DOI:** 10.3390/cancers16162891

**Published:** 2024-08-20

**Authors:** César Miguel Mejía-Barradas, Ana Amador-Martínez, Eleazar Lara-Padilla, Noemí Cárdenas-Rodríguez, Iván Ignacio-Mejía, Valentín Martínez-López, Gabriela Ibañez-Cervantes, Orlando de Jesús Picado-Garcia, Brayan Domínguez, Cindy Bandala

**Affiliations:** 1 Escuela Superior de Medicina, Instituto Politécnico Nacional, Mexico City 11340, Mexico; cmejia@ipn.mx (C.M.M.-B.); vhlp1006@gmail.com (E.L.-P.); gabrielaibanezcervantes@gmail.com (G.I.-C.), braydodope@hotmail.com (B.D.); 2 Departamento de Radiología e Imagen, Centro Médico ABC, Mexico City 01120, Mexico; anaamdormd@gmail.com; 3 Laboratorio de Neurociencias, Instituto Nacional de Pediatria, Mexico City 04530, Mexico; noemicr2001@yahoo.com.mx; 4 Laboratorio de Medicina Traslacional, Escuela Militar de Graduados en Sanidad, Universidad Del Ejército y Fuerza Aérea, Mexico City 11200, Mexico; ivanignacio402@gmail.com; 5 Unidad de Ingeniería de Tejidos, Terapia Celular y Medicina Regenerativa, Instituto Nacional de Rehabilitación Luis Guillermo Ibarra Ibarra, Mexico City 14389, Mexico; val_mart76@yahoo.com.mx; 6 División de Investigación, Hospital Juárez de México, Mexico City 07760, Mexico; 7 Ciudad de la Salud para la Mujer-Clínica Materna Huixquilucan, Huixquilucan de Degollado 52760, Mexico; dr.opicadog@gmail.com

**Keywords:** beta blocker, ADRB, bone mineral density, breast cancer, osteoporosis, osteopenia

## Abstract

**Simple Summary:**

Breast cancer (BCa) is a pathology that affects the world population. Patients present risk factors for osteopenia and osteoporosis, such as chronic stress, estrogen deprivation due to hormonal treatment, a menopausal status, and decreased physical activity. An available alternative is the use of nonselective beta blockers (nsBBs) that have antitumoral properties, and in other diseases, antiresorptive and osteoforming effects. We demonstrated that BCa patients taking nsBBs had a greater bone mineral density and a lower incidence of osteoporosis and osteopenia than untreated patients. Although our study has several limitations, such as its retrospective design and single-center population, it revealed promising effects of nsBBs on the prevention and treatment of osteoporosis and osteopenia in patients with BCa.

**Abstract:**

Breast cancer (BCa) is related to chronic stress and can reduce the bone mineral density (BMD) through neurochemicals related to beta-adrenergic receptor (ADRB) 1 and 2. Selective beta blockers (sBBs) and nonselective beta blockers (nsBBs) are used to treat systemic arterial hypertension (SAH) and may have osteoprotective effects, as they inhibit ADRBs. To evaluate the effects of sBBs and nsBBs on the BMD of Mexican patients with BCa. A retrospective study was conducted. We included 191 Mexican women with BCa without SAH and with SAH treated with nsBBs, sBBs, and diuretics. BMD was evaluated using a bone density scan (DEX scan). A greater average BMD (*p* < 0.05) was observed in patients with prior treatment with both nsBBs and sBBs (0.54 ± 0.94 and −0.44 ± 1.22, respectively) compared to patients treated with diuretics or without SAH (−1.73 ± 0.83 and −1.22 ± 0.98, respectively). Regarding the diagnosis of osteoporosis/osteopenia, no cases were observed in patients treated with nsBBs, whereas 5.6% of the patients treated with sBBs presented osteopenia. A total of 23.1% and 10.6% patients managed with diuretics or without treatment presented with osteoporosis and 61.5% and 48% patients managed with loop diuretics and without treatment presented with osteopenia, respectively (*p* < 0.05). Treatment with nsBBs is a promising option for the prevention and management of osteoporosis/osteopenia in Mexican patients with BCa; however, further prospective studies are needed.

## 1. Introduction

Breast cancer (BCa) is the most common neoplasm in women and the most commonly diagnosed cancer globally [1]. According to the International Agency for Research on Cancer, in the period from 2022 to 2050, an increase in the incidence and mortality of this pathology has been calculated worldwide (from 2.3 to 3.2 and from 0.67 to 1.2, respectively), for Latin America (from 219.7 to 341.9 and from 59.7 to 104.9, respectively), and for Mexico (from 31 to 50.3 and from 8.2 to 15.2, respectively) [2,3]. These statistics highlight the importance of continuing to develop strategies for the prevention, diagnosis, treatment, and promotion of quality of life of patients with BCa.

BCa is characterized by the uncontrolled growth of abnormal cells, where mammary cells undergo numerous genotypic and phenotypic changes [4,5] and may be classified according to many factors, such as size, differentiation, prognosis, method of detection, or genetic alterations. The histological types of BCa are prognostically relevant because they are determined by the specific cells that are affected and by the pathological features of invasiveness [3,6,7].

Contemporary models for BCa treatment include surgery and cytotoxic, hormonal, and targeted therapies in accordance with the molecular profile and clinical stage [8]. Because hormonal receptor-positive BCa is the most prevalent subtype, the use of aromatase inhibitors is considered the standard treatment; however, they reduce estradiol levels, which also increases osteoporosis and fractures [9,10,11].

On the other hand, BCa is often accompanied by psychoemotional conditions, leading to increased chronic stress, which deteriorates the quality of life and increases physical fragility in patients. These alterations, along with hormonal treatment, bone metastasis, and decreased physical activity, are risk factors for a decreased bone mineral density (BMD) [12,13,14,15,16,17]. Chronic stress in BCa patients causes the hypothalamus to secrete corticotropin-releasing hormone and the pituitary gland to release adrenocorticotropic hormone. The adrenal glands then secrete cortisol, inducing the production and secretion of molecules such as catecholamines and glucocorticoids. These molecules influence the homeostasis of bone regulation, causing complications such as fractures, hypocalcemia, and severe pain [18,19]. The catecholamines that are increased in these patients are adrenaline (AD) and noradrenaline (NA) [12,20], both of which are related to tumor progression in BCa patients because they have the potential to induce physiological effects that may lead to changes in immune function and the inflammatory response [15,20,21,22] and an accelerated loss of BMD [12,23,24,25,26]. AD and NA exert their effects through the activation of target molecules known as beta-adrenergic receptors (ADRBs), including both the β1-adrenergic receptor (ADRB1) and the β2-adrenergic receptor (ADRB2) [12]. The most described ADRB in BCa is ADRB2, which has been shown to cause increased proliferation, tumor growth, metastasis, and complications in the bone microenvironment, disrupting the cascade of events that regulate bone resorption [19]. Cells in bone tissue, such as osteoblasts and osteoclasts, also express ADRBs, and their ligands can regulate the homeostasis of BMD [12,27]. Catecholamines, through ADRB2, decrease osteoblastic activity and increase the activity of osteoclasts (bone-resorbing cells), inhibiting the differentiation and adhesion of osteoblasts (bone-forming cells), increasing their level of apoptosis, and delaying collagen synthesis, thus impairing the capacity of osteoblasts to fully replace the resorbed bone [28]. BCa cells can regulate osteoclast activity and act through osteoblasts by stimulating osteoblast-derived receptor activator of NF-κB ligand (RANKL), which is a master regulator of osteoclastogenesis [12,29,30] and reduces the BMD in response to chronic stress [12,19]. Considering the importance of ADRB1 and ADRB2 and their ligands in tumor progression in BCa and in BMD homeostasis, a treatment option for both targets is management with beta blockers (BBs), which are competitive inhibitors of ADRBs. BBs can be selective (sBBs), which block ADRB1, or nonselective (nsBBs), which block both ADRB1 and ADRB2. BBs are particularly known for managing systemic arterial hypertension (SAH) [31]. Different studies have repositioned nsBBs as antitumoral agents in BCa because they inhibit signaling pathways related to metastasis, angiogenesis, chemoresistance, and cell proliferation [32,33,34]. On the other hand, in non-BCa patients or menopausal models, BBs have been reported to inhibit the mRNA expression of osteoclast-related genes by increasing bone formation and decreasing bone resorption, mainly through the inhibition of RANKL expression [19]. In this context, preclinical studies have demonstrated that the use of BBs is associated with an increased BMD. Animal experiments have shown that nsBBs, such as propranolol, prevent bone resorption and promote bone formation by increasing the transverse growth of the endosteum and tibia in rats, increasing bone osteointegration by negatively regulating the number of osteoclasts, positively regulating collagen formation, and promoting mineralization [24,35,36]. In addition, propranolol has been shown to enhance osseointegration and bone volume and regeneration in rats with unicortical defects, improve bone loss and bone fragility in spontaneously hypertensive rats [36,37], and reduce bone resorption in a model of experimental periodontal disease, probably by inhibiting osteoclastogenesis mediated by RANKL and inflammatory markers [38]. Clinical studies have shown that elderly patients with cardiovascular diseases have a reduced risk of osteoporosis and fractures after receiving nsBB treatment [39,40]. The use of sBBs in postmenopausal women was associated with an increased BMD and a low fracture risk [41]. In another study, bone biopsies obtained from elderly women treated with atenolol (50 mg/day) and nebivolol (5 mg/day) had a better bone microarchitecture than did those obtained from placebo-treated individuals, reducing bone resorption and increasing the BMD of the ultradistal radius [42].

The repositioning of BBs for the prevention and treatment of osteoporosis and osteopenia may be a viable alternative for improving the quality of life of patients with BCa. Importantly, the clinical benefits of sBBs or nsBBs on BMD or bone metabolism in BCa patients are still unclear. Therefore, in the present retrospective study, we aimed to evaluate the BMD of Mexican patients with BCa, including women with systemic arterial hypertension (SAH) treated with nsBBs, sBBs, loop diuretics, and those without SAH.

## 2. Materials and Methods

### 2.1. Trial Oversight

An observational and prospective study was performed in Centro Médico ABC, Mexico City. This research was carried out in full accordance with good clinical practice guidelines and was registered with the Research and Ethics Committee of the hospital (TABC-17–22). All patients signed informed consent forms, and their data were confidently protected in accordance with the Mexican General Health Law.

### 2.2. Patients

We included 191 women with a diagnosis of BCa based on clinical data, biopsy, and imaging studies. As the main inclusion criterion, we considered patients with a diagnosis of stage I to IV BCa who were treated for at least 3 months and up to 12 months with an sBB (atenolol 100 mg/day or metoprolol 200 mg/day), nsBB (propranolol 160 mg/day), or a loop diuretic (furosemide 80 mg/day) for the management of SAH, and we also included patients without SAH. We excluded pregnant patients with a diagnosis of chronic kidney disease or thyroid disease, patients with known osteoporosis/osteopenia prior to the diagnosis of BCa, and patients who did not undergo a BMD determination.

### 2.3. Study Procedures and Outcomes

BMD was evaluated with central dual energy X-ray absorptiometry (DXA scan) after obtaining informed consent. The BMD determination was carried out by a single radiology specialist for all patients. DXA scans were performed on the lumbar spine and both coxofemoral joints using a Hologic Densitometer (Discovery WI model) with serial number 87039. Densitometry was classified according to the WHO using the T-score estimator, which represents the number of standard deviations of bone mineral density (sd-BMD) with respect to the mean value of the population aged 20 to 39 years of the same sex: normal ≥ −1.0, osteopenia −1.0 to −2.5, and osteoporosis ≤ −2.5 sd-BMD. Clinical and histological data were obtained from electronic records.

### 2.4. Statistical Analysis

To describe the data, means and standard deviations, frequencies, and percentages were calculated. The distribution type was evaluated using the Kolmogorov–Smirnov test. Student’s *t*-test, analysis of variance (ANOVA), Tukey’s multiple comparison test, chi-square test, and Fisher’s exact test were applied. Due to the retrospective design of the study, we calculated odds ratios and confidence intervals from contingency tables examining the relationship between two dichotomous variables. A *p* value < 0.05 was considered to indicate statistical significance. Analyses were performed using GraphPad Prism software, version 8.0.0 for Windows (GraphPad Software, San Diego, CA, USA), and SPSS software, version 19 (IBM Corp. Released 2015. IBM SPSS Statistics for Windows, version 19.0. Armonk, NY, USA). As control strategies for confounding variables, we carried out a stratified analysis of the main outcomes (age), and homogeneity was demonstrated in relation to clinicopathological characteristics and risk factors for BCa among the study groups.

## 3. Results

### 3.1. Clinical and Demographic Data

A total of 191 patients with a diagnosis of BCa and an age range of 34–83 years were included. Among these patients, 35.6% (68 patients) had a previous diagnosis of SAH; 53% (36/68 patients) were treated with nsBBs, 28% (19/68 patients) with sBBs, and 19% (13/68 patients) were treated with diuretics. No differences were found in risk factors for BCa among the study groups (Table 1).

Table 2 shows the clinicopathological characteristics of patients with BCa in relation to the study group. Significant differences were detected in tumor stage and metastasis at the time of diagnosis (*p* < 0.05 for each variable), with the most aggressive stages and highest frequencies of metastasis observed in patients not treated with nsBBs.

### 3.2. Follow-Up and Outcomes

**Figure 1:**
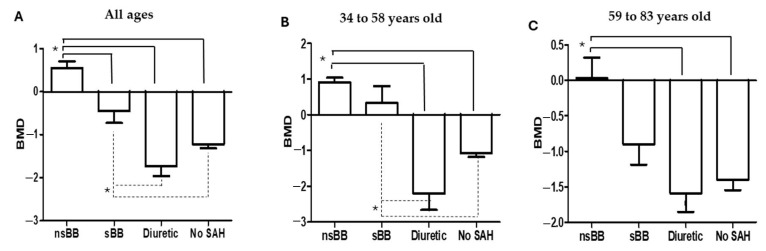
Comparison of the mean bone mineral density between the study groups. Effect of treatment with BBs on bone mineral density in patients with BCa determined by DXA scans. (**A**) All patients, (**B**) patients aged 34 to 58 years, and (**C**) patients aged 59 to 83 years. * *p* < 0.05.

Table 3 shows the mean BMD of patients with BCa in relation to the study group. The values for the total number of patients were considered; however, they were also analyzed by age strata. The mean BMD across all ages of the patients, as well as by age stratum, was greater in those who were treated with nsBBs than in those who were treated with sBBs. The mean BMD was lower in patients treated with loop diuretics than in patients who did not receive any treatment. Figure 1 shows the comparison of mean BMDs between all study groups, highlighting the differences between groups according to the post hoc analysis.

**Figure 2:**
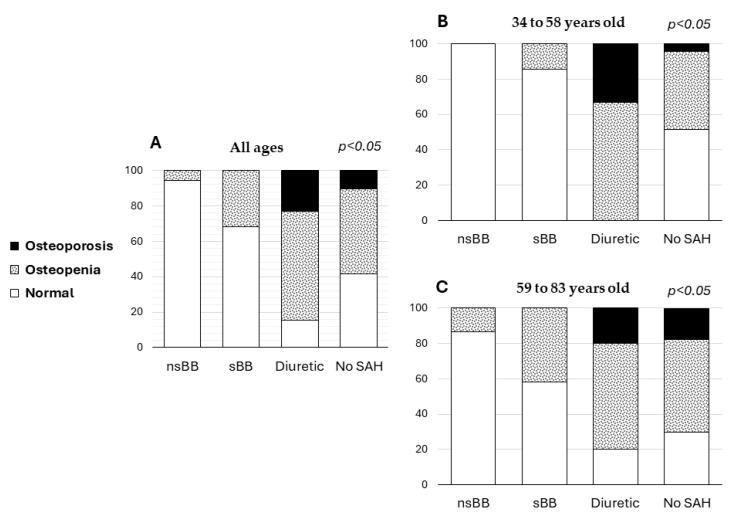
Frequencies of the diagnoses of osteopenia and osteoporosis in the SAH treatment groups and the group without SAH. (**A**) All patients (34 to 83 years old), (**B**) patients aged 34 to 58 years old, and (**C**) patients aged 59 to 83 years old.

According to the BMD classification into normal, osteopenia, and osteoporosis groups, in the group of patients who received treatment with nsBBs or sBBs, no cases of osteoporosis were observed; however, the patients who received diuretic management presented a frequency of 23.1% in the analysis of all ages, whereas 33.2% of the patients in the age group of 34 to 58 years and 20% of the patients in the age group of 59 to 83 years presented osteoporosis. Regarding the diagnosis of osteopenia, frequencies of 5.6% and 31.6% were observed in the group of patients treated with nsBBs and sBBs, respectively, and 61.5% and 48% were observed in the patients managed with loop diuretics and without treatment, respectively, considering all the age groups. This same trend was observed in the analysis by age stratum (Table 4 and Figure 2).

Table 5 shows that all patients with a diagnosis of osteoporosis/osteopenia were 26 times (all ages), 44 times (34 to 58 years old), and 16 times (59 to 83 years old) more likely not to be treated with nsBBs than women treated with nsBBs (*p* < 0.05).

For the group of patients not treated with sBBs, the probabilities of being diagnosed with osteoporosis/osteopenia were 4 times and 5 times greater than those of patients treated with sBBs among all patients and in the group aged 59 to 83 years, respectively (*p* < 0.05).

## 4. Discussion

BCa is associated with bone demineralization due to different pathophysiological mechanisms, such as increased chronic stress, treatment with antiestrogenic and aromatase inhibitors, bone metastasis, and reduced mobility and physical activity [43,44,45,46,47,48,49,50,51]. Recent studies have shown that the link between cancer and chronic stress is mediated by neurochemicals such as cortisol, AD, and NA, which have protumor effects [21,22].

BCa is a neoplasm characterized by the overexpression of ADRBs with high affinity for AD and NA; therefore, management with BBs has shown promise as an antitumor agent [52,53]. This finding is similar to our results, as we observed patients with prior BB treatment had a lower tumor stage and a lower rate of metastasis than patients without a history of this treatment. In this context, various studies have shown chronic stress, mediated by neurochemicals and their ligands, can enhance different protumor mechanisms, such as angiogenesis, metastasis, chemoresistance, and cell proliferation [54]. Chronic stress levels can even increase in patients with neoplasms, leading to post-traumatic stress syndrome, which implies critical stress especially in the first three months after a BCa diagnosis [16,55,56,57,58,59].

For the analysis of BMD in relation to prior BB treatment, stratification was conducted by the age range. We observed a significantly lower BMD in patients aged 59 to 83 years compared to those aged 34 to 58 years. These data are consistent with the previously described negative correlation between age and BMD [46,60,61].

The mean BMD was greater in patients taking nsBBs in both the overall analysis and the age-stratified analysis, followed by those taking sBBs. The lowest BMDs were observed in patients treated with loop diuretics or those without any SAH treatment. The frequencies of osteopenia and osteoporosis were lower in patients treated with BBs, particularly nsBBs. These results can be explained by the antiresorptive effect of BBs, mainly nsBBs, as they prevent osteoclast activation by blocking ADRB1/2 receptors and promote osteoformation by activating osteoblasts through the RANKL pathway [27,28,33,36,55,62]. These findings may be related to the role of C-terminal domain nuclear envelope phosphatase 1 (Ctdnep1) in bone homeostasis, where bone morphogenetic protein (BMP) receptors suppress transforming growth factor (TGF-β) signaling and negatively regulate osteoclast differentiation [12,29,30,48,50,51,63,64]. These results were confirmed by studies of hypertensive rats, where treatment with nsBBs inhibited osteoclast activity and activated osteoblasts through the RANKL pathway [65]. It was also clear that sBBs had a smaller effect, possibly because acting only on ADRB1 and not on ADRB2 results in a smaller effect on RANKL, but this is still unclear [12]. Regarding clinical trials, few studies have evaluated the effects of nsBBs and sBBs on bone metabolism. For example, a clinical trial is investigating the effect of propranolol (Concor^®^, 5 mg once daily for 1 year) on the number of fractures in older adults with osteoporosis [66]. Additionally, the effects of atenolol, nevibolol, and propranolol (50 mg/day, 5 mg/day, and 40 mg b.i.d., respectively, for 20 weeks) on the ratio of serum bone formation to bone resorption markers in postmenopausal women have been studied [67]. Finally, the effects of atenolol (50 mg daily over 2 years) on the percent change in femoral neck and lumbar spine BMD and cortical bone microarchitecture in postmenopausal women have been studied [68]. In contrast to what was reported in the clinical trials, our BCa patients included in this study were treated with higher doses of both nsBBs and sBBs, as the SAH treatment regimens were managed, and the treatment time ranged from 3 months to 1 year, whereas in the aforementioned trials, it was 5 months to a maximum of 2 years. In this sense, although we observed significant results regarding the probable osteoprotective effect of nsBBs, we can mention limitations of our findings; as this was a retrospective study, we did not control the doses and time of treatment with nsBBs, as the regimens were based on the indications for SAH, and so we recommend that clinical trials be carried out where the specific dosage of nsBB is established as osteoprotective both in patients at risk, such as those at menopause, and those with specific pathologies, such as BCa. Importantly, the effects of nsBBs and sBBs on BMD may differ depending on the pathologies of the population, as reported by Anumas et al., who conducted a retrospective study of patients receiving weekly hemodialysis and concluded that patients treated for 5 months with nsBBs had no significant differences in mean BMD compared with untreated patients [69].

On the other hand, the administration of loop diuretics such as furosemide in this study was associated with a decrease in BMD because it promoted the accelerated excretion of urine, calcium, and other minerals important for bone maintenance [70]. However, other studies have shown that diuretics, especially thiazides, which retain calcium, have a protective effect on bone health. We suggest that BCa patients who require a diuretic for SAH treatment be treated with thiazides, as they are more appropriate than loop diuretics are [60,71].

Our results showed that nsBB therapy reduced the probability of a diagnosis of osteoporosis or osteopenia in patients with BCa. The possible mechanisms of action in relation to BMD can be attributed, as previously mentioned, to its antiresorptive and osteoforming capacities, which negatively regulate the number of osteoclasts, positively regulate collagen formation, and promote bone mineralization [8]. Another advantage is that, as an anxiolytic, it reduces the chronic stress experienced by patients with BCa, thereby lowering the levels of cortisol, AD, and NA, which, as already mentioned, precipitate the loss of BMD [72]. Finally, the antitumor effect of BBs limits cancer progression and metastasis [52,53].

The repositioning of nsBBs for the prevention and treatment of osteoporosis and osteopenia is an affordable alternative in terms of cost, availability, and therapeutic safety. Compared with other osteoforming and antiresorptive drugs that can have negative effects on tumor pathology, such as estrogen management or human recombinant parathyroid hormone agents, it can be a first option for preventing and treating bone demineralization and its consequences when they are not severe [73,74,75]. In this context, for BCa patients with osteoporosis and a risk of fracture, combining nsBBs with bisphosphonates such as zoledronic acid or the monoclonal antibody denosumab, which has demonstrated antitumor effects on BCa, would be useful [73,76,77,78,79,80]. For BCa patients with a diagnosis of osteopenia, postmenopausal women, or women at risk of osteoporosis, the prescription of nsBBs with vitamin D could be useful for preventing accelerated bone resorption [81,82,83,84]. However, further prospective studies and clinical trials are needed to confirm these findings.

## 5. Conclusions

In this retrospective study, we demonstrated that Mexican patients with a diagnosis of BCa who received previous treatment for SAH with nsBBs had a greater BMD and no cases of osteoporosis or osteopenia compared to patients treated with sBBs, loop diuretics, or without SAH.

Although our results were obtained from a single-center study, the mechanism underlying our findings could benefit BCa patients from other populations. Prospective studies evaluating specific dosage parameters for the protection of BMD in patients with BCa are needed.

Finally, in patients with BCa, treatment with nsBBs, in addition to the known antitumor benefits, appears promising from the perspective of osteoprotection.

## Figures and Tables

**Table 1:** Risk factors for BCa in relation to the treatment group.

	Study Group				
	nsBB Mean ± SD	sBB Mean ± SD	Diuretics Mean ± SD	No SAH Mean ± SD	*p* Value
Age (range)	56.17 ± 9.1 (41–79)	59.32 ± 9.6 (46–74)	63.54 ± 6.9 (53–79)	57.36 ± 10.9 (34–83)	0.13
Body mass index	24.68 ± 4.3	27.18 ± 7.2	26.57 ± 5.5	25.03 ± 4.3	0.19
	% (n = 36)	% (n = 19)	% (n = 13)	% (n = 123)	
Family history of cancer	50 (18)	26.3 (5)	53.8 (7)	44.7 (55)	0.32
Smoking	33.3 (12)	31.6 (6)	30.8 (4)	37.4 (46)	0.91
Obesity	8.3 (3)	21.1 (4)	15.4 (2)	11.4 (14)	0.55
Early menarche	2.8 (1)	5.3 (1)	7.7 (1)	4.1 (5)	0.88
Nulliparity	5.6 (2)	5.3 (1)	7.7 (1)	5.7 (7)	0.99
Late menopause	0	0	0	1.6 (2)	0.77
Menopause	83.3 (30)	78.9 (15)	100 (13)	79.7 (98)	0.33

SD = standard deviation, nsBB = nonselective beta blocker, sBB = selective beta blocker, SAH = systemic arterial hypertension.

**Table 2:** Clinicopathological characteristics of patients with BCa in relation to the study group.

	Study Group				
	nsBB % (n = 36)	sBB % (n = 19)	Diuretics % (n = 13)	No SAH % (n = 123)	*p* Value
Stage I–II	97.2 (35)	100 (19)	76.9 (10)	77.2 (95)	0.007
Stage III–IV	2.8 (1)	0	23.1 (3)	22.8 (28)	
Triple negative	30.6 (11)	52.6 (10)	23.1 (3)	50.4 (62)	0.05
Triple positive	16.7 (6)	21.1 (4)	15.4 (2)	11.4 (14)	0.64
Hormonal therapy	2.8 (1)	15.8 (3)	0	8.1 (10)	0.23
Metastasis	2.8 (1)	0	7.7 (1)	17.1 (21)	0.02

nsBB = nonselective beta blocker, sBB = selective beta blocker, SAH = systemic arterial hypertension.

**Table 3:** Bone mineral density of patients with BCa in relation to the study group.

	Study Group				
	nsBB Mean ± SD	sBB Mean ± SD	Diuretic Mean ± SD	No SAH Mean ± SD	*p* Value
All patients	+0.54 ± 0.94	−0.44 ± 1.22	−1.73 ± 0.83	−1.22 ± 0.98	0.0001
Age range 34 to 58 years	+0.90 ± 0.59	+0.32 ± 1.24	−2.2 ± 0.80	−1.07 ± 0.82	0.0001
Age range 59 to 83 years	+0.03 ± 1.12	−0.90 ± 0.99	−1.59 ± 0.83	−1.39 ± 1.12	0.0001

SD = standard deviation, nsBB = nonselective beta blocker, sBB = selective beta blocker, SAH = systemic arterial hypertension.

**Table 4:** Diagnoses of osteoporosis/osteopenia in patients with BCA and their relationships with the study group.

	Study Group				
	nsBB % (n = 36)	sBB % (n = 19)	Diuretics % (n = 13)	No SAH % (n = 123)	*p* Value
All patients					
Normal	94.4 (34)	68.4 (13)	15.4 (2)	41.5 (51)	0.0001
Osteopenia	5.6 (2)	31.6 (6)	61.5 (8)	48 (59)	
Osteoporosis	0	0	23.1 (3)	10.6 (13)	
34 to 58 years old	% (n = 21)	% (n = 7)	% (n = 3)	% (n = 66)	
Normal	100 (21)	85.7 (6)	0	51.5 (34)	0.0001
Osteopenia	0	14.3 (1)	66.7 (2)	43.9 (29)	
Osteoporosis	0	0	33.2 (1)	4.5 (3)	
59 to 83 years old	% (n = 15)	% (n = 12)	% (n = 10)	% (n = 57)	
Normal	86.7 (13)	58.3 (7)	20 (2)	29.8 (17)	0.002
Osteopenia	13.3 (2)	41.7 (5)	60 (6)	52.6 (30)	
Osteoporosis	0	0	20 (2)	17.5 (10)	

nsBB = nonselective beta blocker, sBB = selective beta blocker, SAH = systemic arterial hypertension.

**Table 5:** Probability of a diagnosis of osteoporosis/osteopenia in relation to the study group in patients with BCa.

	Osteoporosis/Osteopenia			
	Yes	No	OR (95% CI)	*p* Value
All patients				
No nsBB	97.6% (83/85)	60.9% (53/87)	26.62 (6.13 to 115.5)	0.0001
No sBB	94.6% (70/74)	81.5 (66/81)	3.97 (1.25 to 12.50)	0.01
34–58 years old				
No nsBB	100% (35/35)	61.8% (34/55)	44.25 ^#^ (2.57 to 759.9)	0.0001
No sBB	94.7% (36/38)	86.8% (33/38)	2.72 (0.49 to 15.0)	0.21
59–83 years old				
No nsBB	96% (48/50)	59.4% (19/32)	16.42 (3.38 to 79.77)	0.0001
No sBB	94.4% (34/36)	76.7% (33/43)	5.15 (1.04 to 25.31)	0.02

OR = odds ratio, # = the OR was calculated by adding 0.5 to each value, CI = 95% confidence interval.

## Data Availability

The data presented in this study are available upon request to the corresponding author since they are the clinical data of patients protected under a confidentiality letter delivered to the Ethics Committee of the ABC Medical Center.
